# Effect of Phosphorus Application on Arsenic Species Accumulation and Co-Deposition of Polyphenols in Rice Grain: Phyto and Food Safety Evaluation

**DOI:** 10.3390/plants10020281

**Published:** 2021-02-02

**Authors:** Arghya Chattopadhyay, Anand Prakash Singh, Deepak Kasote, Indrajit Sen, Ahmed Regina

**Affiliations:** 1 Department of Soil Science & Agricultural Chemistry, Institute of Agricultural Sciences, Banaras Hindu University, Varanasi 221005, India; arghya.chattopadhyay1@bhu.ac.in (A.C.); dean_ag@bhu.ac.in (A.P.S.); 2 Centre of Excellence in Rice Value Addition (CERVA), International Rice Research Institute (IRRI)—South Asia Regional Centre (ISARC), Varanasi 221106, India; indraorg@gmail.com

**Keywords:** arsenic speciation, LC-ICP-MS, LC-MS/MS, phosphorus, phenolics, flavonoids, rice, toxicity

## Abstract

The present study was aimed at exploring the effect of soil application of different concentrations of orthophosphate (P) (0, 10, 20, 30, and 40 mg kg^−1^) on rice agronomic and yield parameters, arsenic (As) species accumulation, and polyphenol levels in the grain of rice grown under As spiked soil (10 mg kg^−1^). The contents of As species (As(V), As (III), MMA and DMA) and polyphenols in rice grain samples were estimated using LC-ICP-MS and LC-MS/MS, respectively. P treatments significantly reduced the toxic effects of As on agronomic parameters such as root weight and length, shoot and spike length, straw, and grain yield. Among the treatments studied, only the treatment of 30 mg kg^−1^ P helps to decrease the elevated levels of As (V), As (III), and DMA in rice grains due to As application. The study revealed that 30 mg kg^−1^ was the optimal P application amount to minimize AS accumulation in rice grains and As-linked toxicity on agronomic parameters and chlorophyll biosynthesis. Furthermore, the levels of trans-ferulic acid, chlorogenic acid, caffeic acid, and apigenin-7-glucoside increased in response to accumulation of As in the rice grain. In conclusion, the precise use of phosphorus may help to mitigate arsenic linked phytotoxicity and enhance the food safety aspect of rice grain.

## 1. Introduction

Arsenic (As) is a non-essential element for plants and humans, and it has been found to exhibit considerable toxicity upon excess accumulation [1]. Source of As in soil may be geogenic or anthropogenic [2,3]. Irrigation with As contaminated water further adds the risk of soil contamination in crop fields [4,5]. The elevated level of As in soil is found to influence the yield of crops [6]. Along with a decrease in crop yield, the occurrence of As in the food chain is also a primary concern, considering overall food safety.

Rice, the staple food for half of the world population, is prone to accumulating a considerable amount of inorganic As when grown in As contaminated soils [7]. Flooding of paddy soil leads to reductive dissolution and mobilization of land-bound As into the plant parts [8]. As concentrations in rice grains, husks, stalks, and roots are found to have a positive correlation with their levels in soil and irrigation water [9]. In rice, As is present in several forms, which are mutually interchangeable [1]. The probable toxicity of these forms is in the order of AsH3 > Arsenite (As (III)) > Arsenate (As (V)) > MMA (monomethyl arsonic acid) > DMA (dimethyl arsinic acid) [10]. The rice grain accumulates considerably higher levels of inorganic As than other food grains [11]. The concentration of As above 200 µg Kg^−1^ in polished rice may pose a significant health risk in humans [12].

The exposure of As has an adverse impact on the morphological (reduction in leaf number and chlorosis), physiological (inhibition in overall growth processes, photosynthetic efficiency, and biomass accumulation), and biochemical (oxidative stress and damage of biomolecules) responses of plants [13]. As (V) generates toxicity through the replacement of inorganic phosphorous in the key biochemical processes, while As (III) inhibits the enzymatic action by binding with the sulfhydryl groups in proteins that leads to membrane deterioration and subsequently cell death [13,14]. In plants, absorbed As (V) is converted into As (III) in some parts by an enzyme arsenate reductase. As (III) is stored in the vacuole and is further detoxified [15]. Both As (V) and As (III), including their conversion, are also responsible for the production of reactive oxygen species (ROS), which causes oxidative damage [13,16]. Several agronomic mitigation strategies such as cultivating rice under aerobic conditions, intermittent flooding, use of As-hyperaccumulating plant species in rotation, or in combination and alternate wetting and drying conditions are found to be useful in reducing As input and bioavailability in rice [17]. Moreover, the applications of high doses of phosphorus, iron, and silicon fertilizers are found to reduce the toxic effects of As, significantly [17]. However, in chickpea, Pi was found to increase the uptake of As from soil [18]. Some studies showed that a high amount of Pi application in As rich soil could induce As linked toxicity in plants [19,20].

Plants have effective enzymatic and non-enzymatic antioxidant protection mechanisms to attenuate the toxic effects of ROS [21]. Enzymatic defense systems comprise superoxide dismutase, glutathione peroxidase, catalase, and glutathione reductase. In contrast, the non-enzymatic defense systems include low molecular weight antioxidants such as ascorbic acid, carotenoids, glutathione, proline, phenolic acids, flavonoids, and high molecular weight secondary metabolites like tannins [21,22]. The exposure of As has been reported to disturb the enzymatic antioxidant defense system and decrease non-enzymatic antioxidant ascorbate in the rice seedlings [23]. Recently, treatments with inorganic (As (III), As (V)) and organic (dimethylarsinic acid-DMA) As forms to *Acer platanoides* seedlings were induced phenolic contents in the leaves, which was dependent on the As species and their concentrations [1,24]. However, there is limited insight on the impact of the accumulation of As species in rice grain on the profiles of phenolic acids and flavonoids.

This study aimed to explore the As toxicity mitigation potential of phosphorus through understanding the effects of different concentrations of orthophosphate (P) (10–40 mg kg^−1^) application in soil on As species accumulation in the grain of rice grown under As spiked soil (10 mg kg^−1^). The study also investigated the grain polyphenols profiles of rice grown in As and P spiked soil to identify metabolite markers to be used in breeding for low arsenic accumulating rice. This study enabled determining the optimal P application level for minimizing the As toxicity in rice.

## 2. Results

### 2.1. Agronomic Parameters

The As, and combined As and P treatments showed a considerable impact on various agronomic parameters of rice (Table 1). The As treatment (T0) significantly decreased total chlorophyll, root weight and length, shoot and panicle length, straw, and grain yield compared to untreated control (Table 1). A reduction of nearly 50% in grain yield and 39% in straw yield was caused by As treatment (T0) when compared with the control group (C) plants. Moreover, among the studied combined As and P treatments, only treatment T3 significantly alleviated As linked reduction in total chlorophyll content (Table 1).

### 2.2. Arsenic Species Distribution, Accumulation and Phosphorus Content in Rice Grain

Four As species, namely As (III), As (V), MMA, and DMA, were detected in rice grain (Table 2). Among them, the concentration of MMA was very low, and in some samples, it was below the detection limit. The As application (T0) significantly (*p* < 0.05) increased the levels of As species in grains. Although the applied As was in the form of As(V), we detected a significant increase in all four species in rice grain, indicating the As species conversion occurring in rice. Plants those received 10 mg kg^−1^ As + 30 mg kg^−1^ P (T3 treatment) significantly decreased the elevated concentrations of As (V), As (III), and DMA in rice grains due to the alone As application in soil (Table 2). This finding underlines the critical role of the P application concentration in the paddy field to minimize As accumulation in the rice grain. The T3 (10 mg kg^−1^ As + 30 mg kg^−1^ P) and T4 (10 mg kg^−1^ As + 40 mg kg^−1^ P) treatments showed a significant increase in rice grain P content, compared with P-deficient C and T0 groups. This finding demonstrated that a considerable amount of P was transported to the grain at or above 30 mg kg^−1^ of P application in soil.

### 2.3. Phenolic Acids and Flavonoids Contents in Rice Grain

**Figure 1:**
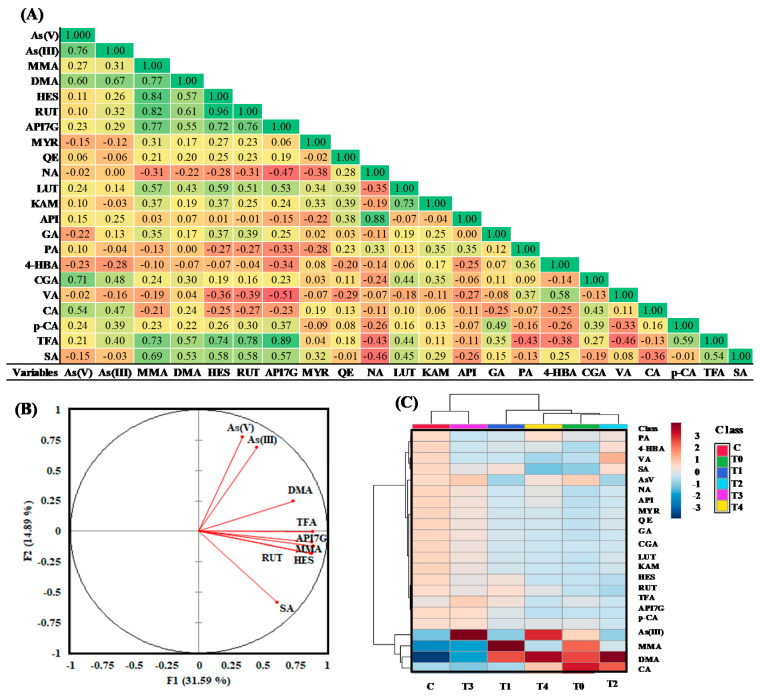
(**A**) Correlation analysis between arsenic species, phenolics, and flavonoids of rice grains. (**B**) Principal component analysis (PCA) biplot plot and (**C**) correlation heatmap, distinguishes different treatment groups based on arsenic species, phenolics, and flavonoids contents of rice grain. Treatments, C and T0, indicate untreated control, and 10 mg kg^−1^ As treated group, respectively. T1, T2, T3, and T4 denote combined treatments, 10 mg kg^−1^ As + 10 mg kg^−1^ P, 10 mg kg^−1^ As + 20 mg kg^−1^ P, 10 mg kg^−1^ As + 30 mg kg^−1^ P, and 10 mg kg^−1^ As + 40 mg kg^−1^ P, respectively.

Amongst the nine phenolic acids analyzed, As treatment (T0) significantly increased (*p* < 0.05) the levels of chlorogenic acid (CGA), caffeic acid (CA), and trans-ferulic acid (TFA) compared with the untreated control (C) (Table 3). No significant changes were found in the levels of gallic acid (GA), 4-hydroxybenzoic acid (4-HBA), protocatechuic acid (PA), *P*-coumaric acid (p-CA), and vanillic acid (VA) in rice grains after As treatment (Table 3). The combined As and P treatments (T1 to T4) also followed the same trend in the case of CGA (Table 3). As-associated increased CA was significantly decreased with the application of P treatments, T1, T2 and T3 compared with T0 treatment (Table 3). Interestingly, As-linked decreased sinapic acid (SA) level was increased in treatments, T1 and T3. Results of correlation studies showed that TFA and SA had the highest positive correction (r = 0.73, and r = 0.69, respectively) with MMA. Similarly, TFA and SA were showing moderate positive correlation with DMA. Interestingly, SA had negative correlation with As (V) and As (III) (Figure 1A,B). Among other phenolic acids, CGA and CA showed moderate positive correlation with As(V) (Figure 1A). 4-HBA showed a negative correlation with all As species. However, the content of HBA among the various treatments studied was not statistically significant.

In addition to phenolic acids, the effects of above different P treatments on flavonoid profiles of rice grain were also studied. Among the nine flavonoids studied, the level of the only apigenin-7-glucoside (API7G) significantly (*p* < 0.05) elevated upon As exposure compared with untreated control (Table 4). The treatment T1, which received 10 mg kg^−1^ of P and As (treatment T1), significantly increased the levels of hesperidin (HES), rutin (RUT), and API7G in rice grain compared with T0 and C groups. However, the higher dose of P application had no further significant impact on HES, RUT, and API7G levels compared with T0 and C treatments. The results of correlation studies showed that HES, RUT, and API7G had the highest positive correction (r = 0.84, r = 0.82, and r = 0.77, respectively) with MMA (Figure 1A). Moreover, HES, RUT, and API7G had moderate positive correction with DMA (Figure 1A). Thus, similar to TFA, API7G could also deposit in response to accumulation of organic As species.

**Figure 2:**
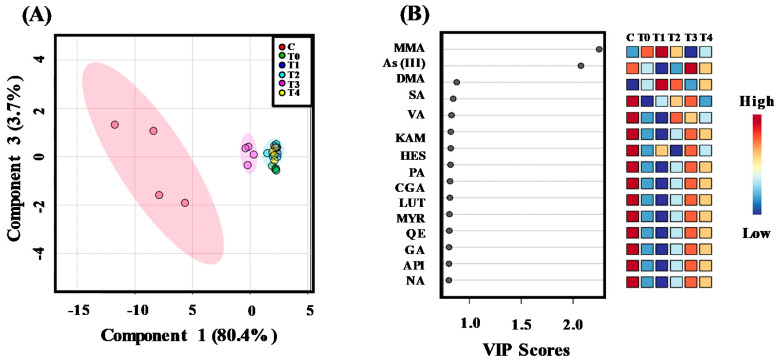
(**A**) Partial least squares discriminant analysis (PLS-DA) scores plot. (**B**) Variable importance in projection (VIP) scores plot indicating the most discriminating arsenic species, phenolics and flavonoids in of rice grain samples before and after exposure of different Arsenic (As) and combined As and phosphate (P) treatments. Treatments, C and T0, indicate untreated control, and 10 mg kg^−1^ As treated group, respectively. T1, T2, T3, and T4 denote combined treatments, 10 mg kg^−1^ As + 10 mg kg^−1^ P, 10 mg kg^−1^ As + 20 mg kg^−1^ P, 10 mg kg^−1^ As + 30 mg kg^−1^ P, and 10 mg kg^−1^ As + 40 mg kg^−1^ P, respectively.

The data were further subjected to multivariant analysis to understand the co-deposition relationship between phenolic compounds and different species of As in rice grain in response to soil application of As and combined As and P treatments. The principal component of analysis (PCA) biplot was constructed to display the relationship among the studied variables (Figure 1B). In PCA biplot, vectors are representing the active variables, and angle between two vectors demonstrating correlation among them. As observed in the correlation matrix study, TFA, HES, RUT, and API7G had a strong positive correlation with MMA. Similarly, SA was also showing positive correlation with MMA and DMA, and negative correlation with As (V) and As (III). Dendrogram from the hierarchical clustering analysis (HCA) was also generated to demonstrate how the studied treatments are grouped, based on the correlation between quantified arsenic species, phenolics, and flavonoids (Figure 1C). We found that all the studied treatments were classified into two main groups. Interestingly, treatment groups, C and T3 were falling under the same cluster, indicating P application at concentration 30 mg kg^−1^ could reduce the accumulation of As in the rice grain. The clustering of all other treatments in dendrogram was further demonstrating P application at a concentration above and below 30 mg kg^−1^ favors As accumulation in rice grain. We performed a supervised dimension reduction analysis, partial least squares discriminant analysis (PLS-DA), to visualize the clustering pattern among various studied treatments, based on arsenic species, phenolics, and flavonoids contents of rice grain (Figure 2A). PLS- DA scores plot explained 81.2% of the total variability of the data (Figure 2A), which is sufficient to interpret the complex interactions existing between treatments and their effects on arsenic species, phenolics, and flavonoids contents of rice grain [25].

In the PLS-DA scores plot, treatments C and T3 only formed distinct individual clusters, while all other treatments clustered together separately. The variable importance on projection (VIP) score plot was derived from the PLS-DA models to identify compounds responsible for clustering based on their VIP score [26]. VIP score plot showed MMA and As (III) had the highest magnitude of VIP scores, respectively (Figure 2B) among the quantified species of As, phenolic acids, and flavonoids. The results of these studies indicate that MMA and As (III) are the main As species of the rice grain, which are highly influenced in response to As and P application.

## 3. Discussion

The phytotoxicity of As is well known. The toxicity of As in plants varies with its speciation, plant species, and with other soil factors controlling As accumulation in plants [1]. In plants, As causes phytoxicity by increasing production of ROS either directly during conversion of As (V) to As (III) or indirectly via inactivation of the enzymatic antioxidants by binding to their thiol group [27]. External phosphate fertilization has been found to be useful in alleviating As toxicity in rice [18,28]. It has been proved that phosphorous is an important parameter in the paddy field for arsenic solubility in soil and its uptake by plants. Both As and phosphorous are the common members of the pnictides group and an analog of each other. Both of these elements have similar charged oxygen atoms, and thermochemical radii with a difference of only 4%, including nearly identical acid dissociation constant values [29]. The competitive adsorption between phosphorous and arsenate occurs in the soil system which is controlled by soil pH, redox potential, binding capacity, interconversion of As species, amounts of phosphorous addition, and rice genotypes [30]. Herein, soil with low available phosphorous (10.3 mg kg^−1^) was used to investigate the role and optimal amount of P in mitigating As induced toxicity in rice. The base As concentration in the soil was low (35 μg kg^−1^) and the phosphorous/As molar ratio was much higher (702.5). In the previous study, As associated toxicity has been reported for lower P/As molar ratios between 3 to 6 [31,32]. In this study, phosphorous/As molar ratio 2.5 was used to induce As toxicity and phosphorous/As molar ratio was further serial increased up to 12 by increasing P amount in the soil to investigate As toxicity mitigation potential of P.

As is also reported to alter the chloroplast formation, including its partial degradation in bean plants [33]. In the chloroplast, As was found to inhibit tetrapyrrole biosynthesis [34]. Tetrapyrrole synthesis plays a major role in retrograde signaling and has a strong interaction with light signaling pathways that are important in chloroplast biogenesis [35]. Results of this study showed that only P treatment at 30 mg Kg^−1^ helps to reverse the adverse effect of As on chlorophyll biosynthesis in rice. Akin to our finding, moderate use of phosphorous fertilizer was found to be useful in improving chlorophyll content in Black Gram (*Vigna mungo* L.Var. PU19) affected by As toxicity [34].

As has adverse effects on the agronomic parameters of plants. In cucumber (*Cucumis sativus* L.), reduction in the xylem sap was observed under As stress, which leads to reduced shoot growth [36]. The toxicity of As on the root and shoot growth as well as on the rice grain yield have been reported in the previous studies [37,38]. Results of this study also demonstrated that As application had detrimental effect on rice growth parameters. In rice, that phosphorous treatment helps to reverse detrimental effect of As on growth, water content and carbohydrate metabolism of the rice seedlings [39]. Similar to these findings, our results also showed that the P treatments with As significantly improved the root weight and length, shoot and panicle length, straw, and grain yield. Moreover, in treatment T3 (10 mg kg^−1^ As + 30 mg kg^−1^ P), the observed As content in grain was below the maximum permissible limit of 0.4 mg kg^−1^, indicating optimal application of P help to improve food safety aspect of rice.

In plants, the protonated neutral forms of As are transported through silicic acid pathway, and As(V) is transported via the phosphate transporter system [40,41]. Because of this, phosphorous and As(V) compete for uptake and translocation in many plants [41]. Interestingly, except As(V), phosphorous does not influence the uptake of other species of As, because of their different transport mechanisms [42]. As(III) is taken up by rice roots via silicic acid transporters [11]. Rice also uptakes methylated species of As such as DMA and MMA but at a much slower rate than inorganic forms of As [9]. Probably because of this, rice grains can accumulate a higher level of inorganic forms of As than organic As [43].

In rice, inorganic and organic As species differ in their mobility from root to shoot. Once, As is taken up by roots, As (V) is reduced to As (III) inside the plant tissues; As (III) is sequestered into root vacuoles or is translocated to the shoots and later disseminated to various organs [44]. In the mobility of As, nodes act as a controlling point, which regulates the As storage and its distribution to the rice grain. However, there is a lot to know about As transport and loading in rice [45]. Phosphorous applications found to have both synergistic and antagonistic impacts on the solubility and bioavailability of As in the soil, which is reported to be mainly dependent on the application concentration of P. Literature reports demonstrated that at low and high phosphorous application concentration, phosphorous may causes phytotoxicity in plants [44]. The excess addition of phosphorousto the arsenic contaminated soil is supposed to enhance the arsenic uptake by rice, *Brassica juncea* L. and *Pteris vittata* [46]. On the contrary, under phosphorous -deficient condition, As adversely effects on the cellular metabolisms due to low pools of phosphorylated primary metabolites and generate toxicity through the replacement of phosphorous from the ATP to form ADP-As [29]. In rice, the accumulated phosphorous influenced the cellular pH, and limits the conversion of As(V) to As (III), and thus lowered the formation of ROS [19]. These observations indicate that the external phosphorous application amount including internal phosphorous level are the critical determinants of As accumulation in the rice. Thus, the optimal application of phosphorous in the root zone is essential for mitigation of As accumulation and its allied toxicity development in plants.

Generally, under stress conditions, both enzymatic and non-enzymatic defense systems of the plants become hyperactive [1]. Antioxidant metabolites such as phenolics help plants to adapt different environmental conditions and in providing protection against oxidative stress [2]. Previous studies demonstrated that As exposure triggers the synthesis of phenolic acids and flavonoids in plants [1,47]. Phenolics act as an antioxidant in plant systems and scavenge ROS, detoxify H_2_O_2_ by donating electrons to guaiacol-type peroxidases [47]. In general, the antioxidant capacity of phenolic compounds is in the order of unsubstituted phenol < monophenols < diphenols < polyphenols [48]. In plants, flavonoids are reported to have lipid peroxidation inhibitory ability [49]. Herein, we estimated 18 phenolic acids and flavonoids in the rice grain samples in control and different treatment groups. Our results showed that the levels of TFA, CGA CA, and API7G were significantly increased in T0 group upon As exposure, which indicates their protective role in mitigating As-associated oxidative stress. Similar to our findings, As stress was reported to induce the TFA level in rice roots [46]. Moreover, intense accumulation of CA and CGA in *Ulmus laevis* Pall leaves is also reported under As stress, especially As (V) [2]. Results of this study also showed a moderate positive correlation with As (V). The lower amount of P application (≤20 mg kg^−1^) significantly increased the levels of SA, HES and RUT, indicating P application influenced the levels of the different phenolic acids and flavonoids of rice grain than those trigged by As exposure alone. Altogether, these findings indicated that TFA, CGA CA and API7G are accumulated in rice grain in response to As stress, and SA can be used as potent marker to distinguish inorganic and organic As species accumulation in rice grain. Moreover, results of present study also indicate the possibility of the production of certain phenolic acid and flavonoid compounds in rice is triggered in response to internal As level, rather than the accumulation of phosphorous. These specific compounds could be useful as biomarkers in breeding programs for developing low As accumulating rice, especially if As dependent increase is detected in vegetative tissues at earlier growth stage, allowing for large scale seedling screening.

## 4. Materials and Methods

### 4.1. Chemicals and Reagents

Authentic standards of phenolic acids (GA, 4--BA, CA, TFA, *p*-CA, VA, and SA), and flavonoids (API7G, HES, kaempferol (KAM), luteolin (LUT), myricetin (MYR), naringenin (NA), quercetin (QE), and RUT) were procured from Sigma-Aldrich, India. The rice flour reference material (NIST1568b) and ammonium formate were also purchased from Sigma-Aldrich, India. Disodium methyl arsonate hexahydrate and dimethylarsinic acid were obtained from Chem Service inc, West Chester, PA, USA. Phenolic acids, CGA and PA, were purchased from HWI Analytik, GmbH, Germany. Apigenin (API) was obtained from Fluka (India). Ultrapure water (resistivity 18.2 M Ω-cm) was obtained from an ultra-pure Lab Q water purification system (Indion Lab Q, India) and used throughout the experiment. Nitric acid (65%–69%, TraceMetal™ Grade) and hydrochloric acid (35%–37%, TraceMetal™ Grade) were purchased from Fisher, India. Standards of arsenic species, As (III) and As (V) were purchased from Supelco, India. Sodium arsenate, potassium dihydrogen phosphate, urea, and potassium chloride were procured from Merck, India. All other chemicals used were of analytical grade.

### 4.2. Net House Experimentation

Healthy paddy (*Oryza sativa* L., variety HUR-105) seedlings were transplanted to cylindrical polythene lined non-perforated earthen pots (diameter 30 cm, height 25 cm) containing 10 kg of alluvial soil. The pots were kept flooded, maintaining 4–5 cm of water above the soil surface, and the experiment was carried out up to four months still the harvesting phase of rice. The base soil level of As and phosphorous were 35 μg Kg^−1^ and 10.3 mg Kg^−1^, respectively and the molar ratio of phosphorous /As was 702.5 (Supplementary Table S1). The pots were placed in a net house, and during the growth period, the mean temperature, evaporation, sunshine hours, relative humidity, and wind speed were 26.13 °C, 2.92 mm, 6.29 h, 87.99%, and 2.75 Km h^−1^, respectively. For the experiment, six groups consisting control (C) and five P treatments (T0–T4) were formed as follows; (1) C-no added As and P; (2) T0only treated with 10 mg kg^−1^ As (dose was selected based on literature reports [50,51] and preliminary observation); (3) T1-treated with 10 mg kg^−1^ As and 10 mg kg^−1^ P; (4) T2-treated with 10 mg kg^−1^ As and 20 mg kg^−1^ P; (5) T3-treated with 10 mg kg^−1^ As and 30 mg kg^−1^ P; and (6) T4-treated with 10 mg kg^−1^ As and 40 mg kg^−1^ P. The calculated amount of As and P were applied as solutions of sodium arsenate (Na_2_HAsO_4_‚7H_2_O) and potassium dihydrogen phosphate (KH_2_PO_4_), respectively. Along with these, 53 mg nitrogen in the form of urea (CO(NH_2_)_2_) in three split and 44 mg potassium in the form of KCl per kg of soil were applied to meet the main nutritional requirement of paddy.

### 4.3. Measurement of Agronomic Parameters and Phosphorus Estimation

Rice plants were harvested by cutting at 5 cm above the soil (to avoid basal tissue contamination by applied arsenate solution). The mass of filled spikelets (i.e., grain including husk) per pot (grain yield) was recorded after freeze-drying the spikelets. Rice grains were separated from their husks using metal-free dehusker, and then grains were ground using mortar and pestle. The straw biomass (defined as the remaining above-ground portion of the rice plant after the spikelets have been removed) was recorded per pot after drying at 65 °C for 72 h. Roots were separated from the soil very carefully using water, and the weight of root biomass per pot was recorded after drying at 65 °C for 72 h. The total chlorophyll contents in the rice leaf samples were estimated using the method described by Hiscox [52]. Phosphorus estimation in rice grain samples was performed as per the protocol described by Jackson [53].

### 4.4. LC–ICP–MS Analysis of Arsenic Species

Rice grain samples were ground into a fine powder using mortar and pestle and sieved through 355 µm (No. 45 ASTM E11) sieve prior to use. Weighed 200 mg powder of each sample and transferred into 50 mL of polypropylene digesting tube for digestion. An aliquot of 10 mL of 1% ultrapure nitric acid was added to the polypropylene tubes containing the samples. The mixture was allowed to stand overnight. Samples were digested in a microwave-accelerated reaction system (Anton Paar, Graz, Austria) using a set program in our laboratory as follows: the temperature was gently raised, first to 55 °C and then to 75 °C, with holding time of 10 min. Finally, the digestion was done at 95 °C with holding time for 30 min. Upon reaching room temperature, samples were centrifuged at 3214× *g* at 4 °C for 5 min using Beckman Coulter Allegra X-30R Centrifuge. The supernatant was collected and again centrifuged at 7168× *g* at 4 °C for 15 min. Samples were kept in the dark on the ice to minimize species transformation until analysis [54]. NIST reference material of rice flour was used to validate the analyses. An aliquot of 50 µL was injected into the Agilent G5654A 1260 HPLC-coupled to an Agilent G8421A ICP-MS. For As speciation, the Agilent ZORBAX SB-Aq (4.6 mm id × 250 mm, 5 μm) reverse phase column was used and maintained at room temperature throughout the analysis. An isocratic mobile phase containing 20 mM citric acid and 5 mM sodium hexane sulphonate, pH 4.3 was used for As species separation. Collision/reaction cell (CRC) mode was used to reduce the polyatomic interference of chloride (Ar35Cl+) in the quantification of 75As.

### 4.5. LC-MS/MS Analysis of Phenolic Acids and Flavonoids

Phenolic acids and flavonoids (soluble-free and -conjugated) were extracted in 80% methanol [55]. Briefly, 1 mL of 80% methanol was added into the microcentrifuge tube containing 50 mg rice flour sample. All tubes were vortexed and sonicated for 10 min and then centrifuged at 10,000× *g* for 10 min at 7 °C. Approximately ~800 μL of supernatant was collected into fresh microcentrifuge tubes, and 1 of mL 80% methanol was added to the residue, followed by the same extraction procedure. The supernatant was again collected in each tube (~1.6 mL) and finally centrifuged at 10,000× *g* for 10 min at 7 °C, and transferred to LC vials for quantitative analysis. Both phenolic acids and flavonoids were analyzed using Agilent 6470 triple-quadrupole mass spectrometer coupled with 1260 Infinity II Prime LC. The separation was carried out on Pursuit 3 PFP (3 µm, 150 × 2 mm) column at the flow rate of 0.43 mL min^−1^ using the gradient mobile phase, 0.1% aqueous formic acid containing 5 mM ammonium formate (A) and 0.1% formic acid in methanol (B). The gradient program for pump B was as follows: 0.0–0.5 min, 2.0%; 0.5–5.0 min, 50%; 5.0–12.0 min, 98%; 12.0–12.1 min, 2%; and 12.1–15.0 min, 2%. The column temperature was set at 40 °C. The mass spectra were acquired in both positive and negative modes using Jet Stream Electrospray Ionization, and quantification of all analytes were carried out in MRM mode. The mass spectrometer was operated under the following conditions: nebulizer gas pressure, 40 bar, nebulizer gas flow, 10 L min^−1^, gas temperature, 300 °C; sheath gas heater temperature, 250 °C, sheath gas flow, 11 L min^−1^, capillary voltage, 4500V and charging voltage, 500 V. Mass Hunter workstation software (Agilent Technologies, USA) was used to control the instruments as well as to acquire and process the data.

### 4.6. Statistics

Results were expressed as a mean ± standard error (SE) of three biological and two technical replicates per sample. SPSS 16.0 was used to conduct one-way ANOVA followed by the post hoc Duncan’s Multiple Range Test (DMRT). XLSTAT (Addinsoft, 2020) and MetaboAnalyst 3.0 (http://www.metaboanalyst.ca/) software were used for multivariate analysis [56].

## 5. Conclusions

Herein, we have demonstrated that the optimal use of P in the soil has a considerable impact on the accumulation of As species and allied toxicity in rice grown under As spiked soil. The findings of this study indicated that the P treatment of concentration 30 mg kg^−1^ was optimal to minimize As linked toxicity on agronomic parameters and chlorophyll biosynthesis, and enhance the safety of rice grain for consumption. Furthermore, TFA, CGA, CA, and API7G were found to deposit in response to accumulation As in rice grain. These phenolic metabolites can be used as biomarkers in low arsenic rice breeding programs to screen out As tolerant seedlings, provided the As dependent increase in these compounds are confirmed in vegetative tissues at the earlier growth period of rice. Moreover, SA can be a potent marker to distinguish inorganic and organic As species accumulation in rice grain. Further studies are envisaged to confirm the expression of these biomarkers at the early vegetative phase of rice growth under As stress conditions. Taken together, this study shows that the precise use of P may help to mitigate arsenic phytotoxicity and enhance the food safety aspect of rice grains.

## Figures and Tables

**Table 1:** Effect of arsenic (As) and combined As and orthphosphate (P) treatments on agronomic parameters of rice (*Oryza sativa* L.). Treatments, C and T0, indicate untreated control, and 10 mg kg^−1^ As treated group, respectively. The base soil As content in control group was 35 μg kg^−1^. T1, T2, T3, and T4 denote combined treatments, 10 mg kg^−1^ As + 10 mg kg^−1^ P, 10 mg kg^−1^ As + 20 mg kg^−1^ P, 10 mg kg^−1^ As + 30 mg kg^−1^ P, and 10 mg kg^−1^ As + 40 mg kg^−1^ P, respectively. Values are expressed as a mean ± standard error (SE) of three biological replicates. Significant differences (*p* < 0.05) among different treatments are shown by different letters.

	Total Chlorophyll (mg g^−1^ F.W.)	Shoot Length (cm)	Root Weight (g)	Root Length (cm)	Spike Length (cm)	Test Weight (g)	Grain Yield (g pot^−1^)	Straw Yield (g pot^−1^)
C	2.48 ± 0.11 a	87.8 ± 1.15 a	42.1 ± 1.79 a	27.5 ± 2.18 ab	28.3 ± 0.79 a	20.4 ± 0.29 a	20.1 ± 0.66 a	44.6 ± 1.43 a
T0	1.83 ± 0.11 c	77.2 ± 1.07 b	22.1 ± 1.07 c	18.3 ± 1.30 c	22.0 ± 0.48 d	20.0 ± 0.15 a	10.2 ± 0.89 c	32.0 ± 1.10 d
T1	2.01 ± 0.17 bc	84.9 ± 1.57 a	33.9 ± 1.84 b	24.3 ± 1.43 b	23.8 ± 0.80 cd	20.0 ± 0.17 a	16.1 ± 0.86 b	36.9 ± 1.52 c
T2	2.14 ± 0.14 a–c	85.7 ± 1.87 a	36.7 ± 1.66 ab	27.0 ± 1.30 ab	26.4 ± 0.94 ab	20.6 ± 0.32 a	17.3 ± 0.51 b	35.9 ± 1.05 cd
T3	2.40 ± 0.12 ab	85.4 ± 1.30 a	41.2 ± 2.09 a	29.7 ±1.13 a	27.5 ± 0.53 ab	20.7 ± 0.15 a	15.7 ± 0.52 b	41.9 ± 1.70 ab
T4	1.86 ± 0.17 c	83.1 ± 1.44 a	37.7 ± 1.76 ab	27.0 ± 1.73 ab	25.5 ± 0.87 bc	20.1 ± 0.15 a	16.7 ± 1.20 b	40.0 ± 1.23 bc

**Table 2:** The contents of arsenic (As) species and orthphosphate (P) in rice (*Oryza sativa* L.) grain after exposure of As and combined As and P treatments. Treatments, C and T0, indicate untreated control, and 10 mg kg^−1^ As treated group, respectively. The base soil As content in control group was 35 μg kg^−1^. T1, T2, T3, and T4 denote combined treatments, 10 mg kg^−1^ As + 10 mg kg^−1^ P, 10 mg kg^−1^ As + 20 mg kg^−1^ P, 10 mg kg^−1^ As + 30 mg kg^−1^ P, and 10 mg kg^−1^ As + 40 mg kg^−1^ P, respectively. Results were expressed as a mean ± standard error (SE) of three biological and two technical replicates per sample. Significant differences (*p* < 0.05) among different treatments are shown by different letters. As (III), arsenite; As (V), arsenate; MMA, monomethyl arsonic acid; DMA, dimethyl arsenic acid.

Treatments	Concentration in µg Kg^−1^					Phosphorus (g Kg^−1^)
	As (V)	MMA	As (III)	DMA	Total As	
C	60.1 ± 8.28 d	nd	23.3 ± 2.91 d	10.0 ± 1.59 e	93.4 ± 12.76 e	2.87 ± 0.20 d
T0	185 ± 17.2 a	1.95 ± 0.03 b	130 ± 9.41 a	363 ± 2.96 b	679 ± 23.6 b	3.07 ± 0.20 cd
T1	125 ± 13.4 bc	6.53 ± 0.08 a	104 ± 7.56 a–c	543 ± 3.34 a	778 ± 11.2 a	3.33 ± 0.24 b–d
T2	120 ± 1.54 bc	0.46 ± 0.13 c	96 ± 17.8 bc	370 ± 19.6 b	586 ± 35.9 c	3.80 ± 0.24 a–c
T3	96.2 ± 7.96 c	nd	83 ± 9.12 c	89.7 ± 1.95 d	269 ± 1.65 d	4.10 ± 0.29 ab
T4	137 ± 8.69 b	0.38 ± 0.04 c	119 ± 2.69 ab	295 ± 8.69 c	551 ± 5.24 c	4.30 ± 0.27 a

**Table 3:** Phenolic acid profiles of rice grain samples after exposure of different Arsenic (As) and combined As and orthphosphate (P) treatments. Treatments, C and T0, indicate untreated control, and 10 mg kg^−1^ As treated group, respectively. The base soil As content in control group was 35 μg kg^−1^. T1, T2, T3, and T4 denote combined treatments, 10 mg kg^−1^ As + 10 mg kg^−1^ P, 10 mg kg^−1^ As + 20 mg kg^−1^ P, 10 mg kg^−1^ As + 30 mg kg^−1^ P, and 10 mg kg^−1^ As + 40 mg kg^−1^ P, respectively. All values are expressed as a mean ± standard error (SE) of three biological and two technical replicates per sample. Significant differences (*p* < 0.05) among different treatments are shown by different letters.

Treatments	Concentration in mg Kg^−1^								
	GA	PA	4-HBA	CGA	VA	CA	p-CA	TFA	SA
C	0.54 ± 0.01 a	0.32 ± 0.10 a	0.94 ± 0.15 a	0.06 ± 0.02 b	0.82 ± 0.06 ab	1.06 ± 0.57 bc	1.02 ± 0.15 a	2.70 ± 0.72 c	0.38 ± 0.01 c
T0	0.56 ± 0.03 a	0.34 ± 0.07 a	0.60 ± 0.12 a	0.09 ± 0.01 a	0.80 ± 0.20 ab	6.37 ± 0.59 a	1.38 ± 0.21 a	4.20 ± 0.32 b	0.29 ± 0.03 c
T1	0.60 ± 0.04 a	0.25 ± 0.02 a	0.79 ± 0.20 a	0.08 ± 0.01 ab	0.65 ± 0.20 b	0.24 ± 0.02 c	1.26 ± 0.15 a	5.80 ± 0.03 a	0.71 ± 0.03 a
T2	0.53 ± 0.02 a	0.32 ± 0.09 a	1.01 ± 0.25 a	0.08 ± 0.01 ab	1.42 ± 0.39 a	4.94 ± 1.17 a	1.04 ± 0.11 a	3.23 ± 0.63 bc	0.53 ± 0.01 b
T3	0.56 ± 0.04 a	0.23 ± 0.03 a	0.69 ± 0.13 a	0.08 ± 0.01 ab	0.66 ± 0.08 b	1.04 ± 0.58 bc	1.22 ± 0.08 a	4.10 ± 0.27 b	0.37 ± 0.07 c
T4	0.54 ± 0.01 a	0.34 ± 0.06 a	0.81 ± 0.18 a	0.08 ± 0.01 ab	0.66 ± 0.03 b	2.64 ± 0.92 b	1.18 ± 0.09 a	3.25 ± 0.15 bc	0.27 ± 0.04 c

**Table 4:** Flavonoid profiles of rice grain samples after exposure of different Arsenic (As) and combined As and orthphosphate (P) treatments. Treatments, C and T0, indicate untreated control, and 10 mg kg^−1^ As treated group, respectively. The base soil As content in control group was 35 μg kg^−1^. T1, T2, T3, and T4 denote combined treatments, 10 mg kg^−1^ As + 10 mg kg^−1^ P, 10 mg kg^−1^ As + 20 mg kg^−1^ P, 10 mg kg^−1^ As + 30 mg kg^−1^ P, and 10 mg kg^−1^ As + 40 mg kg^−1^ P, respectively. All values are expressed as a mean ± standard error (SE) of three biological and two technical replicates per sample. Significantly differences (*p* < 0.05) among different treatments are shown by different letters.

Treatments	Concentration in mg Kg^−1^								
	HES	RUT	API7G	MYR	QE	NA	LUT	KAM	API
C	0.16 ± 0.01 b	0.11 ± 0.01 b.	0.06 ± 0.01 c	1.74 ± 0.01 a	0.57 ± 0.01 a	0.13 ± 0.01 ab	0.25 ± 0.01 ab	0.15 ± 0.01 ab	0.23 ± 0.01 b
T0	0.19 ± 0.01 b	0.14 ± 0.01 b	0.08 ± 0.01 b	1.73 ± 0.01 ab	0.57 ± 0.01 a	0.12 ± 0.01 b	0.26 ± 0.01 a	0.16 ± 0.01 a	0.24 ± 0.01 b
T1	0.29 ± 0.03 a	0.26 ± 0.03 a	0.09 ± 0.01 a	1.73 ± 0.01 a	0.57 ± 0.01 a	0.12 ± 0.01 b	0.26 ± 0.01 a	0.15 ± 0.01 ab	0.24 ± 0.01 ab
T2	0.14 ± 0.02 b	0.11 ± 0.02 b	0.07 ± 0.01 bc	1.73 ± 0.01 ab	0.57 ± 0.01 a	0.12 ± 0.01 b	0.25 ± 0.01 ab	0.15 ± 0.01 ab	0.23 ± 0.01 b
T3	0.16 ± 0.01 b	0.13 ± 0.01 b	0.08 ± 0.01 b	1.71 ± 0.01 b	0.57 ± 0.01 a	0.13 ± 0.02 ab	0.25 ± 0.01 b	0.15 ± 0.01 ab	0.24 ± 0.01 ab
T4	0.17 ± 0.02 b	0.15 ± 0.02 b	0.07 ± 0.01 bc	1.72 ± 0.01 ab	0.57 ± 0.01 a	0.14 ± 0.01 a	0.25 ± 0.01 b	0.14 ± 0.01 b	0.26 ± 0.02 a

## Data Availability

The data presented in this study are available on request from the corresponding author.

## Supplementary Materials

The following are available online at https://www.mdpi.com/2223-7747/10/2/281/s1, Table S1: Physical, chemical and biological properties of initial soil.
