# Supplementary material for: Effect of Phosphorus Application on Arsenic Species Accumulation and Co-Deposition of Polyphenols in Rice Grain: Phyto and Food Safety Evaluation
Source: Plants (Basel). 2021 Feb 2;10(2):281. doi: 10.3390/plants10020281 (PMC7912781; doi:10.3390/plants10020281)
Supplement: Supplementary file 1 [file plants-10-00281-s001.pdf]

Supplementary information:

Effect of Phosphorus Application on Arsenic Species Accumulation and Co-deposition of Polyphenols in Rice Grain: Phyto and Food safety Evaluation

Arghya Chattopadhyay [1,†], Anand Prakash Singh[1], Deepak Kasote [2,†,*], Indrajit Sen[2] and Ahmed Regina[2,*]

[1] Department of Soil Science & Agricultural Chemistry, Institute of Agricultural Sciences, Banaras Hindu University, Varanasi-221005, Uttar Pradesh (U.P.), India; arghya.chattopadhyay1@bhu.ac.in (A.C); apsinghbhu@rediffmail.com (A.P.S.)

[2] Centre of Excellence in Rice Value Addition (CERVA), International Rice Research Institute (IRRI) - South Asia Regional Centre (ISARC), Varanasi-221106, Uttar Pradesh (U.P.), d.kasote@irri.org (D.K.); indraorg@gmail.com (I.S.); a.regina@irri.org (A.R.)

***** Correspondence: a.regina@irri.org (A.R.); d.kasote@irri.org (D.K.); Tel.: +91-542-2518900.

† These authors contributed equally to this work.

Table S1. Physical, chemical and biological properties of initial soil.

**Soil properties**	**Values**
Sand (%)	62.7
Silt (%)	20.7
Clay (%)	16.6
Texture	Sandy-clay-loam
Family	Typic Ustochrept
p^H	7.28
EC (dSm^{-1})	0.3
BD ($Mg\ m^{-3}$)	1.32
PD ($Mg\ m^{-3}$)	2.65
WHC (%)	44.8
OC ($g\ kg^{-1}$)	3.42
CEC ($meq\ Kg^{-1}$)	0.98
OC ($g\ kg^{-1}$)	3.42
Available N ($mg\ Kg^{-1}$)	108
Available P_2O_5 ($mg\ Kg^{-1}$)	10.3
Available K ($mg\ Kg^{-1}$)	137
Available As ($\mu g\ K^{-1}$)	35
